# Low dose irradiation profoundly affects transcriptome and microRNAme in rat mammary gland tissues

**DOI:** 10.18632/oncoscience.94

**Published:** 2014-11-10

**Authors:** Lidia Luzhna, Olga Kovalchuk

**Affiliations:** ^1^ Department of Biological Sciences, University of Lethbridge, Lethbridge, Canada

**Keywords:** mammary gland, low dose radiation, gene expression, microRNAome

## Abstract

Ionizing radiation has been successfully used in medical tests and treatment therapies for a variety of medical conditions. However, patients and health-care workers are greatly concerned about overexposure to medical ionizing radiation and possible cancer induction due to frequent mammographies and/or CT scans. Diagnostic imaging involves the use of low doses of ionizing radiation, and its potential carcinogenic role creates a cancer risk concern for exposed individuals. In this study, the effects of X-ray exposure of different doses on the gene expression patterns and the micro-RNA expression patterns in normal breast tissue were investigated in rats. Our results revealed the activation of immune response pathways upon low dose of radiation exposure. These included natural killer mediated cytotoxicity pathways, antigen processing and presentation pathways, chemokine signaling pathways, and T- and B-cell receptor signaling pathways. Both high and low doses of radiation led to miRNA expression alterations. Increased expression of miR-34a may be linked to cell cycle arrest and apoptosis. Up-regulation of miR-34a was correlated with down-regulation of its target E2F3 and up-regulation of p53. This data suggests that ionizing radiation at specific high and low doses leads to cell cycle arrest and a possible initiation of apoptosis.

## INTRODUCTION

Ionizing radiation is a powerful tool in medical diagnostics and the most successful cancer treatment after surgery. The major difference in the use of ionizing radiation between diagnostic procedures and radiation therapy is the applied radiation dose. High doses of radiation possess cytotoxic properties required to kill tumor cells [1]. Diagnostic imaging, on the other hand, involves the use of low doses of ionizing radiation to gather the necessary information about a disease without harmful side effects [2]. However, a potential carcinogenic role of ionizing radiation creates a cancer risk concern for exposed individuals. The biological effects of low doses and dose rates of radiation on normal tissues have been the subject of intense research and discussion [3]. According to the Linear-Non-Threshold (LNT) model, low-dose and low-dose-rate exposure results in a similar cancer risk as high-dose exposure [4]. On the other hand, the LNT model is frequently challenged by the hormetic effect theory according to which low doses of radiation may make the exposed cells less susceptible to later high-dose exposure and may have health benefits [5].

Microarray technology for gene expression analysis may provide a better understanding of biological effects of low doses of ionizing radiation. The radiation response at the gene expression level can help reveal the mechanisms of cellular response and identify key genes responsible for specific endpoints [6]. There are only a few published *in vivo* studies focusing on gene expression analysis in tissues exposed to low doses of ionizing radiation. A clear distinction between high and low doses of gamma radiation has been shown in the liver tissue of mice [7]. Similar effects of low and high doses of radiation have been found in the thymus tissue of mice, with 2421 and 608 genes being affected after high and low doses, respectively [8]. A different response has been shown for internal low-dose radiation from ^131^I. The response of transcripts has been found to be independent of a dose but rather tissue dependent [3]. Overall, there is no clear evidence of an exact mechanism of radiation response at the gene expression level, especially in *in vivo* models. Some reasons might be tedious animal handling, the heterogeneity of the absorbed dose, a mixture of cell types within a tissue, among others.

Gene expression is strongly regulated by epigenetic modifications, including negative regulation of protein synthesis by microRNAs. Ionizing radiation causes alterations in miRNA expression and subsequently, in protein levels of key regulators of the cell cycle. For instance, 2.5 Gy of X-rays caused upregulation of miR-34a and downregulation of miR-7 in hematopoietic tissues [9]. Targets for miR-34a are oncogenes myc, notch1, e2f3, and cyclinD1; miR-7 targets a regulator of DNA methylation, a lymphoid-specific helicase (LSH). The differential expression of miRNAs in response to different doses of gamma radiation was observed previously in human B lymphoblastic (IM9) cells. Low-dose (0.5 Gy) irradiated cells showed a decrease in onco-miRNAs - miR-20 and 21, while high-dose irradiation (10 Gy) caused upregulation of miR-197 that can stimulate carcinogenesis [10]. It was hypothesized that low doses of irradiation suppressed carcinogenesis, while high doses could promote it, and these effects would be miRNA-mediated.

The aim of this study was to investigate the effects of different doses of X-ray exposure on gene expression patterns and micro-RNA expression patterns in normal rat breast tissues.

## RESULTS

### The effects of low, intermediate, and high doses of radiation on whole-genome gene expression in the mammary gland

**Figure 1:**
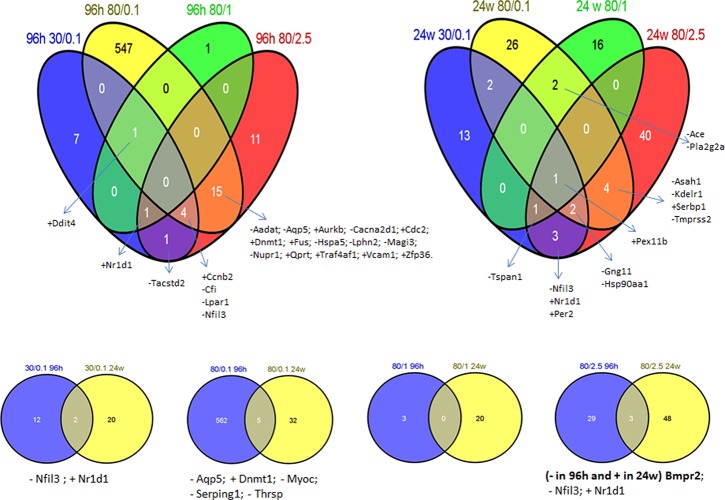
Differentially expressed genes commonly shared between treatment groups The Venn diagram groups the common altered genes between experimental groups.

Isolated RNA from the mammary gland was used for gene expression profiling. A drastic difference in the radiation-induced gene expression changes was discovered between the doses/energy levels applied. Ninety-six hours after radiation, only high energy level/low doses of X-ray exposure (80kVp/0.1 Gy) led to significant alterations in the expression level of 567 genes (Table 1). Other doses did not affect gene expression, and only a few genes were altered. Interestingly, the alterations noticed at an early time point disappeared by 24 hours, while the slight (51 genes) delayed gene expression alterations were noticed for the high level/high doses (80kVp/2.5 Gy) of radiation (Table 1). Most of the altered genes were unique in their experimental groups, and there were not many genes common to all the treatment groups (Fig. 1).

**Table 1:** Gene expression profiling in mammary gland tissue exposed to low and high doses of ionizing radiation The number of significantly changed genes in the rat mammary gland upon low energy level/low dose (30kVp/0.1 Gy), high energy level/low dose (80kVp/0.1 Gy), high energy level/medium dose (80kVp/1 Gy) and high energy level/high dose (80 kVp/2.5 Gy) of radiation in comparison to their corresponding un-irradiated controls at 96 hours and 24 weeks time points, as identified by the gene expression profiling analysis.

Treatment Group	96 hours			24 weeks		
	Total number of genes changed	Number of up-regulated genes	Number of down-regulated genes	Total number of genes changed	Number of up-regulated genes	Number of down-regulated genes
**30 kVp/0.1 Gy**	14	8	6	22	8	14
**80 kVp/0.1 Gy**	567	295	272	37	10	27
**80 kVp/1 Gy**	3	3	0	20	7	13
**80 kVp/2.5 Gy**	32	13	19	51	28	23

**Figure 2:**
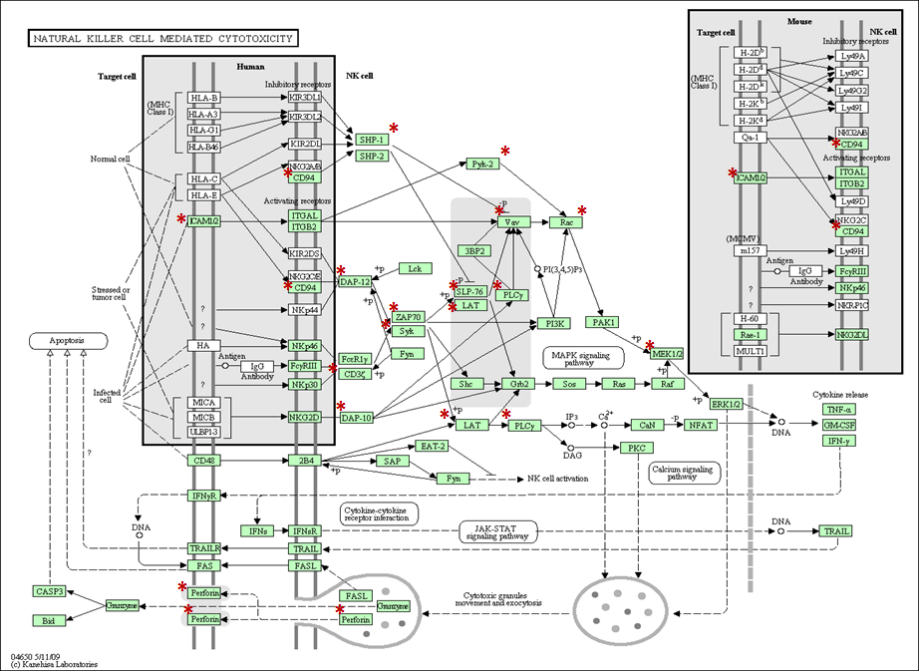
The KEGG Natural Killer Cell Mediated Cytotoxicity Pathway The red stars represent genes that were upregulated.

Further, we evaluated 567 genes that changed their expression level 96 hours after 80kVp/0.1 Gy of X-rays: 295 genes were upregulated, and 272 genes were downregulated. With the help of the DAVID functional annotation array analysis tools, we were able to identify and group the evaluated genes according to their function and possible role in certain pathways. Subsequently, genes with a similar or identical function were grouped together; and based on their expression changes, the role of certain pathways in radiation response was evaluated (Table 2). Most of the changed genes contributed to certain immunological pathways (Table 2). Some examples of such elevated pathways are as follows: antigen processing and presentation (16 genes altered), B- and T-cell receptor signaling (13 and 15 genes, respectively), chemokine signaling (14 genes), Fc gamma R-mediated phagocytosis (11 genes), natural killer cell-mediated cytotoxicity (18 genes), etc. Upregulation of immunological pathways reveals the activation of immune defense against possible damages caused by either ionizing radiation or other forms of potential stressors. The visual representation of one of such pathways (natural killer cell-mediated cytotoxicity) is presented in Figure 2. Most downregulated genes contributed to metabolic pathways: citrate cycle (8 genes), fatty acid metabolism (6 genes), glutathione metabolism (7 genes), pyruvate and tryptophan metabolism (7 and 6 genes, respectively) (Table 2). The number of altered genes in the 24-week/80kVp/2.5 Gy group was too small to group them in the pathways; therefore, we analyzed singular genes of interest.

**Table 2:** Significantly altered KEGG pathways in mammary gland upon 96h of 80kVp/0.1 Gy in comparison to the corresponding un-treated controls In this table, the pathway significance (%) is defined as the ratio of gene alterations that similarly affect a certain pathway (either up- or down-regulate) to the total number of altered genes in the pathway. “+” – the pathway is up-regulated; “-” – the pathway is down-regulated.

Pathways	Pathway Significance, % (total number of genes)
Antigen processing and presentation	+ 93.8% (16)
B cell receptor signaling	+ 100% (13)
Cell adhesion molecules (CAM)	+ N/S (20)
Chemokine signaling	+ 100% (14)
Citrate cycle (TCA)	− 100% (8)
Cytosolic DNA-sensing pathway	+ 100% (6)
ECM-receptor interaction	− 88.9% (9)
Fatty acid metabolism	− 100% (6)
Fc epsilon RI signaling	+ 100% (10)
Fc gamma R-mediated phagocytosis	+ 100% (11)
Glutathione metabolism	− 85.7% (7)
Graft-vs-host disease	+ 100% (7)
Hematopoietic cell lineage	+ N/S (9)
Intestinal immune network for IgA production	+ 100% (6)
Leukocyte transendothelial migration	+ 89.5% (19)
Lysosome	+ N/S (13)
Natural killer cell mediated cytotoxicity	+ 100% (18)
PPAR signaling	− 100% (10)
Primary immunodeficiency	+ 100% (8)
Pyruvate metabolism	− 100% (7)
T cell receptor signaling	+ 100% (15)
Tryptophan metabolism	− 83.3 % (6)

**Figure 3:**
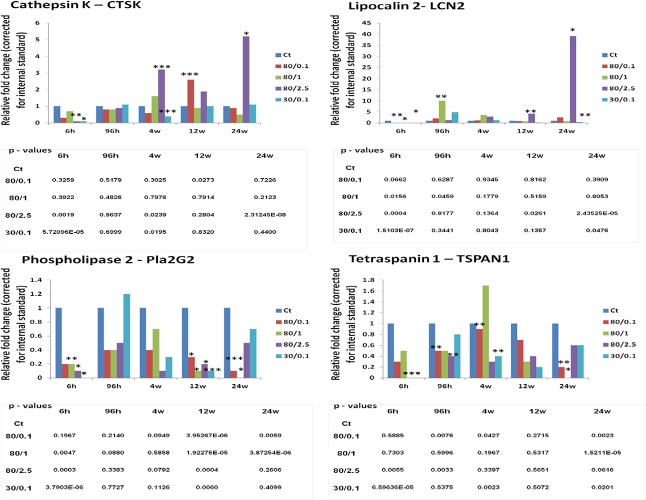
The altered levels of gene transcripts of cathepsin K, lipocalin 2, phospholipase 2, and tetraspanin 1 as detected by RT-PCR The data are shown as fold changes to respective controls. Each treatment group was compared to its corresponding control; B-actin was used as a reference gene (calculated by Pfaffl). P-values (in the tables below the graphs) were calculated by Student's t-test.

The validity of gene expression profiling was confirmed by qRT-PCR for genes with the most change and the greatest radiation response in both the 96 hours/80kVp/0.1 Gy and 24 weeks/80kVp/2.5 Gy groups. Therefore, the primary targets for qRT-PCR were cathepsin K (CTSK), lipocalin 2 (LCN2), phospholipase 2 (Pla2G2), and tetraspanin 1 (TSPAN1) (Fig. 3). CathepsinK, a lysosomal cysteine proteinase that was known to be overexpressed in breast cancers, was significantly elevated at 4 and 24 weeks after high-dose radiation (80kVp/2.5 Gy). Lipocalin 2, an oncogene that may function as a growth factor, was also upregulated at 24 weeks after the highest dose of X-rays. Both phospholipase 2 and tetraspanin 1 play a role in cell growth, signaling and motility. Similarly to the gene expression analysis, qRT-PCR showed that these genes were downregulated in most experimental groups at 24 weeks after radiation exposure (Fig. 3).

### miRNA expression in the irradiated mammary gland

miRNAs regulate gene expression epigenetically; therefore, we proceeded to analyze the role of miRNAs in response to low, intermediate, and high doses of radiation in mammary gland tissue at 96 hours after treatment. miRNAs involve the epigenetic control of gene expression regulation through the RNA interference pathway. miRNAs negatively affect the levels of their target transcripts and proteins encoded by these transcripts. In this way, miRNAs contribute to gene silencing, and changes in miRNA expression are common in cancers and in response to radiation.

Interestingly, we identified the alterations in miRNA expression after high dose/energy level (80 kVp/2.5 Gy) and low dose/low energy level (30kVp/0.1 Gy) radiation (Table 3). Upregulation of miR-34a has been found to be common for both doses, and the expression level has been increased 1.55- and 1.08-fold after 80 kVp/2.5 Gy and 30 kVp/0.1 Gy, respectively. MiR-34a directly inhibits the expression of transcription factor E2F3 that is necessary for cell progression through cell cycle and the expression of actin cross-linking protein, transgelin, which may contribute to the replicative senescence. The MiR-34 family is known to be activated by the p53-dependant pathway in response to DNA damage.

**Table 3:** Radiation-induced microRNA expression changes in rat mammary gland Relative miR expression values are represented in folds in the irradiated cells in comparison to non-irradiated control cells as analyzed by miRNA microarray. Significance of differences was analyzed by the Student's t-test.

Treatment Group	MiRNA changed	Log2 (G/CT)	Validated targets
**80 kVp/0.1 Gy**	2	Low fold change	–
**80 kVp/0.1 Gy**	Low signals	–	–
**80 kVp/1 Gy**	miR-34a	1.55	E2F3, Tagln, INHBB
	miR-29c	−1.02	Tpm1
	miR-20b-5p	−1.65	–
	miR-204	−1.39	–
**30 kVp/2.5 Gy**	miR-34a	1.08	E2F3, Tagln, INHBB
	miR-20b-5p	−1.55	–
	miR-98	−1.16	–
	miR-127	2.08	–

### Tp53, E2F3, and transgelin expression in the irradiated mammary gland

**Figure 4:**
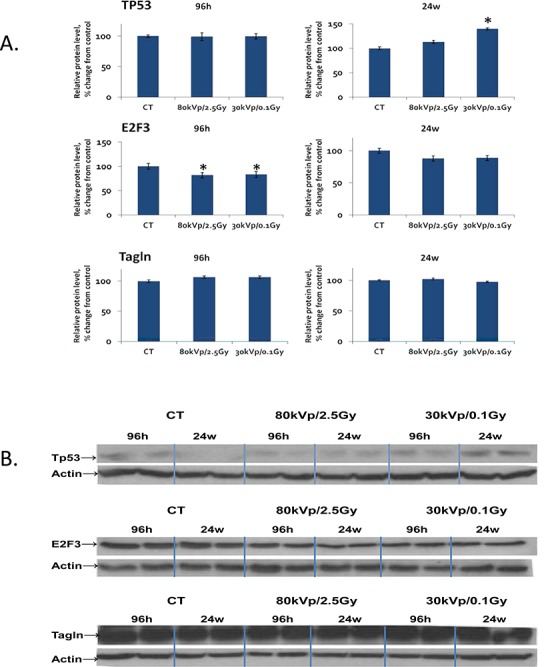
The levels of Tp53, E2F3 and transgelin protein in the rat mammary gland upon whole-body irradiation Protein levels relative to those of control non-irradiated animals are shown as Mean ± St Er. Representative blots are shown of two independent experiments. * - p < 0.05, the Student's t-test.

The elevated expression of miR-34a was interesting to us, and we decided to proceed with identifying protein levels of its targets E2F3 and transgelin as well as p53, the key protein in DNA damage response. Western analysiswas performed for tissues exposed to 80 kVp/2.5 Gy and 30kVp/0.1 Gy radiation, at 96 hours and 24 weeks after exposure. The level of Tp53 was shown to be significantly elevated at 24 hours after low-dose exposure (Fig. 4). The increased levels of the phosphorylated p53 protein stimulated radiation response and DNA damage repair.

The level of E2F3 protein was shown to be decreased in response to both 80kVp/2.5 Gy and 30kVp/0.1 Gy radiation treatments at early time point (96 hours) (Fig. 4). The downregulation of E2F3 is known to stimulate G1 arrest, senescence, and/or apoptosis. There were no significant differences in the expression of transgelin in the irradiated tissues in comparison to non-irradiated controls (Fig. 4).

## DISCUSSION

Ionizing radiation has been successfully used inmedical tests and treatment modalities for a variety of medical conditions, including breast cancer screening and therapy. Nevertheless, a strong concern about overexposure to medical ionizing radiation and possible cancer induction due to continuous mammography procedures and/or CT scans exists amongst patients and individuals who provide patient care [4]. The raised concern is based on the ability of low doses of ionizing radiation used for diagnostic procedures to cause DNA damages that are not extensive enough to induce cell death but may result in mutations, genomic rearrangements and cancer initiation [17]. Ionizing radiation is considered to be a non-threshold carcinogen. The Linear-No-Threshold (LNT) model states that there is no dose level below which radiation exposure is safe, and there is a finite probability that even the lowest possible dose may be responsible for cancer initiation [4]. It is evident that choosing the right dose of radiation as well as the amount of radiation used during screening and therapy is vital for any medical procedure to minimize any potential risk of harm. Overall, the data on the response of healthy mammary tissues to low versus high doses and energy levels of radiation are scarce and indeed need more experimental evidence.

In the present study, the immediate (96 hours) and prolonged (24 weeks) radiation-induced changes in mammary gland gene expression were investigated and compared between different radiation doses and energy levels. Unexpectedly, the large-scale gene expression alterations were only noticed after the application of high energy/low dose (80kVp/0.1 Gy) X-rays at 96 hours after treatment (Table 1). Neither high-dose nor low-dose exposures combined with low-energy radiation caused significant modifications in gene expression at the transcription level. The altered genes mainly constituted the immunological pathways that were shown to be activated upon radiation (Table 2). Radiation is generally considered to be an immunosuppressive agent that kills radiosensitive cells, and this makes radiotherapy one of the most successful cancer therapies. However, under certain circumstances, especially exposure to low-dose radiation may enhance immunity. Our study has shown an increase in antigen processing and presentation, aprocess by which antigen-presenting cells digest foreign proteins and display antigenic peptide fragments on MHC molecules for the recognition by T cells during infections and abnormal cell growth. Among the genes that were upregulated and contribute to this pathway were the following: CD74 (the major histocompatibility complex class two that plays a role in MHCII antigen processing), CD8a (involved in T cell-mediated killing by identifying cytotoxic T cells that interact with MHC class I), Ifi30 (the interferon gamma inducible protein that facilitates MHC class I and II recognition of antigens containing disulfide bonds), and other genes with similar functions. A similar effect of radiation on antigen presentation by MHC class I was reported previously in murine colon adenocarcinoma cells [18]. Similarly, non-cytotoxic effects of ionizing radiation on MHC class I antigen presentation were demonstrated in bone marrow-derived dendritic cells [19]. The modulation of antigen presentation pathways provides protective anti-tumor immunity to the irradiated cells and tissues. Eighteen genes constituting the natural killer (NK) cell-mediated cytotoxicity pathway were also upregulated (Table 2, Fig. 2). NK cells play a role in immune surveillance for cancer by providing anticancer immunity to cells [20]. The activated genes were CD247 (it plays a role in signal transduction upon antigen triggering), Icam 1 and 2 (they are ligands for leukocyte adhesion), lat (a linker for T activation), among others. The enhancement of NK cell-mediated cytotoxicity after radiation in combination with HDAC inhibitor was recently reported in lung cancer cells [21]. B- and T-cell receptor signaling pathways were also upregulated upon low dose/high energy radiation (Table 2). Both pathways stimulate immune response to cancer initiation and are the prime targets for the treatment of many malignancies. Various chemoattractants for blood monocytes and memory T-helper cells as well as chemokine receptor genes were activated; this activation upregulated the chemokine signaling pathway (Table 2). Similar CXC chemokines were shown to be upregulated by extremely low doses of ionizing radiation in normal human fibroblasts [22]. The upregulation of the phogocytosis pathway was due to an increased expression of 11 genes. Phagocytosis activation has been known to be induced after radiation exposure as a consequence of recognition and clearance of radiation-induced apoptotic cells. Such inflammatory-type response to radiation exhibits the bystander effect of radiation rather than the direct effect of radiation [23]. Overall, the activation of immune response pathways upon radiation exposure may indicate anti-tumor protection and eradication of damaged cells. Similar effects of internal low-dose irradiation on gene expression and activation of immune response in normal tissues in mice were reported previously [3]. Interestingly, immune response was the only common biological process affected by irradiation in all tissues studied (the liver, lung, spleen, kidney medulla, and kidney cortex), while alterations in other biological processes were tissue-specific [3].

Radiation response has also shown metabolic changes, mainly downregulation of citrate cycle, pyruvate, and fatty acid metabolism pathways (Table 2). A metabolic response to radiotherapy is very important. A progressive decrease in glucose metabolism in cancer has been shown to be useful for the prediction of radiotherapy response [24]. Metabolic properties of pre-cancerous and cancer cells depend on glycolisis, increased fatty acids synthesis, and increased rates of glutamine metabolism. These properties often result in therapeutic resistance [25]. Our results on gene expression have demonstrated the radiation-induced metabolic inhibition that may lead to cell death rather than cancer initiation. Gene expression analysis was confirmed by qRT-PCR for four genes with the highest changes in gene expression. Tetraspanin 1 RNA expression was proved to be decreased upon ionizing radiation (Fig. 3). This protein mediates signal transduction events that play a role in the regulation of cell development, activation, growth, and motility. Phospholipase 2 was down-regulated at the early and late time points, and was thought to participate in the regulation of phospholipid metabolism in biomembranes, including eicosanoid biosynthesis. Phospholipases are ubiquitously expressed and have diverse biological functions, including the roles in inflammation, cell growth, signaling and death, and the maintenance of membrane phospholipids. Interestingly, both gene expression and qRT-PCR analyses have shown an increased expression of lipocalin 2 and cathepsin K 24 weeks after being exposed to the highest dose (80kVp/2.5 Gy). Both proteins are known to be oncogenes and are ubiquitously expressed in breast cancers. It is important to note that high expression of these genes was not accompanied by the upregulation of particular pathways to which these genes belong.

It is well accepted that gene expression is strongly regulated by epigenetic factors [26]. A number of studies have indicated substantial alterations of epigenetic elements, including changes in DNA methylation, histone modification, and short RNA patterns as a result of radiation exposure [27]. Radiation-induced changes in miRNA expression usually lead to changes in the synthesis of proteins involved in the main cellular biological pathways. As per Table 3, the validated targets of misregulated miRNAs fall in cell cycle and apoptosis categories (Table 3). Interestingly, a low radiation dose causes similar miRNA expression changes to the highest dose. The increased expression of miR-34a may be linked to cell cycle arrest and apoptosis. The ectopic expression of miR-34 genes is known to cause a G1 phase arrest [28]. Furthermore, the high expression of miR-34a has been shown to induce apoptosis [29]. The main targets of miR-34a are E2F3 transcription factor, transgelin, and possibly CDK4/6, cyclin E2, c-myc [30]. Bommer et al. showed that Bcl-2 was targeted by miR-34a [31]. Interestingly, several reports have shown that the miR-34 family is a direct target of p53, and its activation induces apoptosis and cell cycle arrest [31, 32]. In addition, the activation of miR34-a by p53 feeds back to p53, and such positive feedback leads to further activation of p53 [30]. We further decided to conduct Western blot analysis to identify protein levels of E2F3 and transgelin that are targets of p53 and miR-34a targets, E2F3 and transgelin. The expression level of E2F3 protein was indeed downregulated at 96 hours after radiation treatment with both low and high doses (Fig. 4). E2F3 binds specifically to RB1 and is involved in the control of cell cycle progression from G1 to S phase. Low levels of E2F3 lead to cell cycle arrest in response to DNA damages that result from ionizing radiation. We did not notice any significant changes in the protein level of transgelin. However, an elevated level of p53 protein was detected after exposure to a low dose of ionizing radiation. Such correlation between upregulated miR-34a, the downregulation of its target E2F3, and the upregulation of p53 allows us to suggest that ionizing radiation at specific high and low doses leads to cell cycle arrest and a possible initiation of apoptosis. The induction of cell cycle arrest and promotion of apoptosis when the damage is too severe to be repaired are considered to be important for tumor suppression [33]. In his report, Hermeking described the role of p53 as a mediator of tumor suppression through the activation of miR-34 family members.

Overall, both post radiation gene expression and miRNA expression analyses have demonstrated an increased immunological response and cell cycle arrest directed to prevent cancer initiation. However, these characteristics were not detected for every dose applied. Further investigation of the cellular response may shed more light on the correlation between differential radiation doses and their effects on apoptosis/cancer.

## MATERIALS AND METHODS

### Animal models and irradiation conditions

Six-week-old intact female Long-Evans rats were obtained from Charles River (Wilmington, MA). The animals were housed two per cage in a temperature-controlled (24 °C) room in a 12-hour light-dark cycle and given *ad libitum* access to water and an NIH-31 pelleted diet. Six rats were randomly assigned to one of the following X-ray radiation treatment groups: 80kVp/0.1 Gy, 80kVp/1 Gy, 80kVp/2.5 Gy, 30kVp/0.1 Gy, and sham treated controls. Each group of animals was humanely sacrificed 6, 96 hours, and 4, 12, and 24 weeks after radiation treatment. The paired caudal inguinal mammary glands were excised. Tissue was frozen immediately in liquid nitrogen and stored at −80°C for subsequent analyses.

### RNA isolation

Total RNA was isolated using the Illustra RNAspin Mini kit (GE Healthcare Life Sciences, Buckinghamshire, UK). Approximately 50–70 mg of mammary gland tissue was processed following the manufacturer's instructions. The samples were eluted in Ultrapure DNase/RNase-free distilled water provided in the kit. RNA samples were quantified by ultraviolet spectroscopy (NanoDrop, Wilmington, DE) and were further assessed for RNA integrity (RIN) on the Aglient 2100 Bioanalyzer (Santa Clara, CA) using the RNA Nano-chip Kit. RNA samples with RIN values of seven or better were followed through to analysis.

### Whole-genome gene expression profiling

### Library preparation

For this study, cRNA was created using the Ambion Illumina TotalPrep RNA Amplification Kit (Applied Biosystems, Carlsbad, CA), with an input of 500 ng of total RNA per sample. Briefly, oligo-dT primers were used to synthesize first-strand cDNA containing a phage T7 promoter sequence. The single-stranded cDNA was converted into a double-stranded DNA template via DNA polymerase. RNase H acted simultaneously to degrade RNA, and cDNA samples were purified in filter cartridges to remove excess RNA, primers, enzymes, and salts. The recovered cDNA was subjected to *in vitro* transcription using biotinylated UTPs. This step created the labeled and amplified cRNA. A final purification step removed unincorporated NTPs, salts, inorganic phosphates, and enzymes to prepare samples for hybridization.

### Hybridization and detection

The Illumina's direct hybridization assay kit was used to process samples according to the manufacturer's protocol (Illumina, San Diego, CA). Briefly, 750 ng from each cRNA sample was hybridized to the Illumina Rat-Ref-12 Whole Genome Expression BeadChip arrays overnight. Afterward, a 10-minute incubation with the supplied wash buffer at 55°C preceded a 5-minute room-temperature wash. The arrays were incubated in 100% ethanol for 10 minutes. A second room temperature wash for two minutes with gentle shaking completed this high stringency wash step. The arrays were blocked with buffer for 10 minutes and washed before a 10-minute probing with steptavidin-Cy3 (1:1000). After a five-minute wash at room temperature, BeadChips were dried and imaged. Six controls were also built into the Whole-Genome Gene Expression Direct Hybridization Assay system to cover the aspects of array experiments. These included controls for a biological specimen (14 probes for housekeeping controls), three controls for hybridization (six probes for Cy3-labeled hybridization, four probes for low stringency hybridization, one probe for high stringency hybridization), signal generation (two probes for biotin control) and ~800 probes for negative controls on an eight-sample BeadChip. The arrays were scanned on the iScan platform (Illumina), and the data were normalized and scrutinized using Illumina BeadStudio software.

### BeadChip statistical analysis and data processing

The false discovery rate (FDR) was controlled by the Benjamini-Hochberg method. The Illumina Custom Model took FDR into account and was used to analyze the data. Differential gene expression (at least a 0.5-fold change) from sham-treated animals was determined to be statistically significant if the p-value after the adjustment with the Benjamini-Hochberg method was less than 0.05. The values were transformed to show a log2 scale.

Lists of regulated transcripts were put into the web-based DAVID Bioinformatics Resources 6.7 (NIAID/NIH) Functional Annotation Tool [11, 12]. This program was used to group genes into functionally relevant categories and pathways for further analysis of the association of genetic profiles with breast cancer susceptibility. The minimum number of genes in each altered pathway was set to three. The pathways were deemed significantly altered if at least 80% of the genes were shifting the pathway in the same direction [13].

### Real-time polymerase chain reaction (qRT-PCR)

Quantitative real-time PCR was performed to confirm the Whole-Genome Gene Expression results for the regulation and direction (either up or down) of the selected genes. Four genes (Cathepsin K, Lipocalin 2, Phospholipase 2, and Tetraspanin 1) were selected from the gene list of significantly differentially expressed transcripts that represented a preliminary review of the acquired gene expression data. *β*-*Actin* was used as a reference gene. All reactions were performed using cDNA synthesized from 500 ng of RNA sample using the Bio-Rad iScript Select cDNA Synthesis Kit (Bio-Rad Laboratories, Hercules, CA). The samples were stored at −20°C for long-term storage and at 4°C until they were used for subsequent qRT-PCR reactions.

The primers were designed using the NCBI database and PrimerQuest (Integrated DNA Technologies, Inc., Coralville, IA). The primers were as follows: *CTSK* forward primer 5′-ATG TGC AGC AGA ATG GAG GCA TTG-3′ and reverse primer 5′-TGC TCT CTT CAG GGC TTT CTC GTT-3′; *LCN2* forward primer 5′ -ACA ACG TCA CTT CCA TCC TCG TCA- 3′ and reverse primer 5′ -TGG CAA ACT GGT CGT AGT CAG TGT- 3′; *PLA2G2A* forward primer 5′ -CAT GGC CTT TGG CTC AAT TCA GGT- 3′ and reverse primer 5′ -ACA GTC ATG AGT CAC ACA GCA CCA- 3′; *TSPAN* forward primer 5′ -TTG TCA ACG TGG GCT ACT TCC TCA- 3′ and reverse primer 5′ -AGC ACA CAC TTG TTC TCG GAG TGA- 3′; and *beta-Actin* reference gene forward primer 5′-CCT CTG AAC CCT AAG GCC AA-3′ and reverse primer 5′-AGC CTG GAT GGC TAC GTA CA-3′. Reactions were prepared using 1 L of diluted cDNA, 10 pmol/L of each forward and reverse primer and Ssofast EvaGreen Supermix (Bio-Rad Laboratories, Hercules, CA) according to the manufacturer's instructions. Samples were prepared in triplicate and were run on the Bio-Rad C1000 Thermal Cycler equipped with the CFX96 Real-Time System. The qRT-PCR protocol consisted of denaturation at 95°C for two minutes; 43 cycles of denaturation (95°C, five seconds) and annealing/extension (55C, five seconds); and the final extension at 65°C for five seconds. For every set of primers, annealing temperature optimization, melting curve analysis, and gel analysis of amplicon were performed. To evaluate PCR efficiency, the standard curve was established using series of cDNA dilutions. The data were captured and organized by the Bio-Rad CFX Manager 2.1 software (Bio-Rad Laboratories, Hercules, CA).

The quantification data from the Bio-Rad CFX Manager software were analyzed in Microsoft Excel using the Pfaffl method [14]. The graphs showing fold change from the sham group were created showing transcript regulation directions (up- or down regulation).

### miRNA microarray expression analysis

Total RNA from mammary gland frozen tissues was isolated using Trizol reagent (Invitrogen, Burlington, ON) according to the manufacturer's instructions. One ug of the total extracted RNA represented as two repeats per experimental group was sent to LC Sciences (Austin, TX) for miRNA microarray analysis.

### Western immunoblotting

For protein isolation, 30–50 mg of mammary gland tissue were washed in PBS, lysed, and sonicated in 0.25 mL of 1% sodium dodecyl sulfate (SDS) containing protein inhibitors. The lysates were cleared using centrifugation. The protein content was determined using the Bradford protein determination assay (BioRad, Hercules, CA). Equal amounts of lysate protein were subsequently run on 10–12% SDS-polyacrylamide gels and transferred to PVDF membranes (GE Healthcare, Baied'Urfé, Québec).

Western immunoblotting was conducted using the well-established protocols [15, 16]. The membranes were incubated with antibodies against mouse anti-TP53, rabbit anti-transgelin, rabbit anti-E2F3 (1:100 dilution, Santa Cruz Biotechnology, Inc., Santa Cruz, CA ), and mouse anti-Actin (1:1000 dilution, Abcam Inc., Cambridge, MA). Antibody binding was revealed through the incubation with horseradish peroxidase-conjugated secondary antibodies (GE Healthcare, Piscataway, NJ) and the ECL Plus immunoblotting detection system (GE Healthcare, Piscataway, NJ). Chemiluminescence was detected using BioMax MR films (Eastman Kodak, New Haven, CT). The unaltered PVDF membranes were stained with Coomassie Blue (BioRad, Hercules, CA) to prove equal protein loading. Signals were quantified using NIH ImageJ 1.63 software and normalized to loading controls. The images are representative of two independent immunoblots. The results are presented as mean ± S.E.M. The statistical analyses were conducted using the student's t-test. P-values less than 0.05 were considered significant
